# Association of Maternal Thyroglobulin With Gestational Thyroid Function and Offspring IQ and Brain Morphology

**DOI:** 10.1210/clinem/dgae679

**Published:** 2024-09-27

**Authors:** Tessa A Mulder, Mònica Guxens, Maria Luisa Rebagliato, Mariana Dineva, Sarah C Bath, Sandra Hunziker, Jordi Sunyer, Juana Maria Delgado-Saborit, Amaia Irizar Loibide, Nerea Lertxundi, Ryan L Muetzel, Henning Tiemeier, Robin P Peeters, Tim I M Korevaar

**Affiliations:** Generation R Study Group, Erasmus University Medical Center, 3015 GD, Rotterdam, The Netherlands; Department of Internal Medicine, Academic Center for Thyroid Diseases, Erasmus University Medical Center, 3015 GD, Rotterdam, The Netherlands; Department of Child and Adolescent Psychiatry, Erasmus University Medical Center, 3015 GD, Rotterdam, The Netherlands; Department of Child and Adolescent Psychiatry, Erasmus University Medical Center, 3015 GD, Rotterdam, The Netherlands; Spanish Consortium for Research on Epidemiology and Public Health (CIBERESP), Instituto de Salud Carlos III, 28029, Madrid, Spain; ISGlobal, 08003, Barcelona, Spain; Department of Medicine and Life Sciences, Universitat Pompeu Fabra, 08002, Barcelona, Spain; Spanish Consortium for Research on Epidemiology and Public Health (CIBERESP), Instituto de Salud Carlos III, 28029, Madrid, Spain; Epidemiology and Environmental Health Joint Research Unit, FISABIO-Universitat Jaume I-Universitat de València, 46020, Valencia, Spain; Department of Medicine, Faculty of Health Sciences, Universitat Jaume I, 12006, Castellón de la Plana, Spain; School of Food Science and Nutrition, Faculty of Environment, University of Leeds, LS2 9JT, Leeds, UK; Leeds Institute for Data Analytics (LIDA), University of Leeds, LS2 9JT, Leeds, UK; Department of Nutrition, Food and Exercise Sciences, Faculty of Health and Medical Sciences, University of Surrey, GU2 7XH, Guildford, UK; Human Nutrition Laboratory, Institute of Food, Nutrition, and Health, ETH Zurich, 8092, Zurich, Switzerland; Institute of Social and Preventive Medicine, University of Bern, 3012, Bern, Switzerland; Spanish Consortium for Research on Epidemiology and Public Health (CIBERESP), Instituto de Salud Carlos III, 28029, Madrid, Spain; ISGlobal, 08003, Barcelona, Spain; Department of Medicine and Life Sciences, Universitat Pompeu Fabra, 08002, Barcelona, Spain; Epidemiology and Environmental Health Joint Research Unit, FISABIO-Universitat Jaume I-Universitat de València, 46020, Valencia, Spain; Department of Medicine, Faculty of Health Sciences, Universitat Jaume I, 12006, Castellón de la Plana, Spain; Spanish Consortium for Research on Epidemiology and Public Health (CIBERESP), Instituto de Salud Carlos III, 28029, Madrid, Spain; Biodonostia Health Research Institute, Group of Environmental Epidemiology and Child Development, 20014, San Sebastian, Spain; Department of Preventive Medicine and Public Health, University of the Basque Country (UPV/EHU), 48940, Leioa, Bizkaia, Spain; Spanish Consortium for Research on Epidemiology and Public Health (CIBERESP), Instituto de Salud Carlos III, 28029, Madrid, Spain; Environmental Epidemiology and Child Development Group, Biogipuzkoa Health Research Institute, 20014, San Sebastian, Spain; School of Psychology, University of the Basque Country, UPV/EHU, 20008, San Sebastián, Spain; Department of Child and Adolescent Psychiatry, Erasmus University Medical Center, 3015 GD, Rotterdam, The Netherlands; Department of Radiology and Nuclear Medicine, Erasmus University Medical Centre, 3015 GD, Rotterdam, The Netherlands; Department of Child and Adolescent Psychiatry, Erasmus University Medical Center, 3015 GD, Rotterdam, The Netherlands; Department of Social and Behavioral Sciences, Harvard T.H. Chan School of Public Health, Boston, MA 02115, USA; Department of Internal Medicine, Academic Center for Thyroid Diseases, Erasmus University Medical Center, 3015 GD, Rotterdam, The Netherlands; Department of Internal Medicine, Academic Center for Thyroid Diseases, Erasmus University Medical Center, 3015 GD, Rotterdam, The Netherlands; Division of Vascular Medicine and Pharmacology, Department of Internal Medicine, Erasmus MC, 3015 CN, Rotterdam, The Netherlands

**Keywords:** thyroid function, thyroglobulin, iodine, brain development

## Abstract

**Background:**

Low maternal urinary iodine concentration (UIC) during pregnancy is associated with adverse offspring neurodevelopment. Thyroglobulin (Tg) has been suggested as a more sensitive biomarker than UIC of long-term iodine status, but associations of Tg with neurodevelopment and the possible mediating role of thyroid function remain unknown.

**Aim:**

To study whether maternal Tg is associated with (1) maternal and newborn thyroid function and (2) offspring IQ and brain morphology.

**Methods:**

Participants were selected from 2 population-based prospective cohorts: Generation R (the Netherlands, iodine-sufficient) and INfancia y Medio Ambiente (Spain, mildly iodine-deficient) with maternal Tg and thyroid function data in the first half of pregnancy or in cord blood, early childhood IQ (age 4.5 and 6 years), late childhood IQ (age 9 and 13), or brain morphology at 10 years. Associations of Tg with TSH, free T4 (FT4), IQ, and brain morphology were studied with multivariable linear regression.

**Results:**

(1) Tg was associated with lower TSH (−0.12 [−0.16; −0.08]) and higher FT4 (0.08 [0.05; 0.12]) in pregnancy (n = 4367) but not with cord blood TSH or FT4 (n = 2008). (2) Tg was associated with lower IQ in early childhood (β [95% confidence interval]: −0.06 [−0.10; −0.01], n = 2919) but not with IQ (n = 2503) or brain morphology (n = 1180) in later childhood. None of the associations of Tg with the studied outcomes differed by the iodine-to-creatinine ratio (ie, effect modification) or changed when adjusted for thyroid function.

**Conclusion:**

Higher Tg is associated with lower IQ in early childhood and higher thyroid function during pregnancy but not with IQ or brain morphology in later childhood. Further research should determine the value of Tg in addition to UIC for defining iodine status.

Iodine is crucial for the production of thyroid hormone, an important regulator of fetal brain development and the metabolic demand during pregnancy (1, 2). Severe maternal iodine deficiency during pregnancy results in maternal hypothyroidism and congenital iodine deficiency disorders (historically known as cretinism), which include severe cognitive impairment (1). Iodine deficiency is recognized as the primary preventable cause of brain damage worldwide (3). Although successful supplementation strategies have practically eliminated severe iodine deficiency, at least one-third of European countries and 20% of pregnant women worldwide are mild to moderately iodine deficient (4, 5).

Research in the last decade indicates that mild to moderate iodine deficiency during gestation can also negatively affect child neurodevelopment (6). Lower maternal urinary iodine-to-creatinine ratio (UI/Creat) before and during pregnancy was associated with a lower offspring IQ (7, 8). Furthermore, findings of 2 studies (which partially overlap with the current study sample) suggest nonlinear associations of UI/Creat with verbal IQ (9) and gray matter volume (10), indicating an optimal range of iodine status for neurodevelopment.

Urinary iodine concentration (UIC) is recommended for the assessment of population iodine status (3). It has also been used as a measure of exposure to study the effects of iodine on neurodevelopment. However, it is well known that UIC is only a proxy for short-term iodine intake with a high intraindividual and day-to-day variability. This can be reduced by creatinine adjustment, but UIC or UI/Creat poorly reflects thyroidal iodine stores (11). This might explain why several studies investigating the relationship of UIC with thyroid function during pregnancy did not find an association (12-15). However, it is biologically highly plausible that any effects of maternal iodine availability on child neurodevelopment outcomes would be mediated via changes in maternal and/or fetal thyroid function.

Although it has been postulated that thyroglobulin (Tg) may be a more sensitive biomarker of long-term iodine status and thyroidal iodine availability, data to support the added value of Tg to UIC for iodine status assessment remain limited (16). Previous studies showed a U-shaped association of UIC with Tg in children and nonpregnant adults (17-19), suggesting that Tg and UIC might be useful as complementary measurements to verify low or high iodine status.

Interestingly, during pregnancy there seems to be a linear association, where only iodine deficiency but not excess was associated with a higher Tg (20). In addition, some studies reported associations between maternal Tg and thyroid function during pregnancy, but data are scarce (21, 22). However, no study has investigated the association of maternal Tg with offspring neurodevelopment.

The main aim of this study was to assess the association of maternal Tg with offspring IQ and brain morphology and the possible mediating role of maternal thyroid function. To support this aim, we first assessed the association of maternal Tg with maternal thyroid function. In addition, we examined the previously studied association of UI/Creat with Tg (23) and the added value of Tg to UI/Creat by assessing if associations of Tg with thyroid function and neurodevelopment were modified by UI/Creat (ie, stronger associations when UI/Creat is low) or changed when adjusted for UI/Creat.

## Methods

### Study Design and Participants

This study was embedded in 2 prospective birth cohorts: Generation R (The Netherlands) and Infancia y Medio Ambiente (INMA, Spain). In Generation R, all pregnant women with an expected delivery date between April 2002 and January 2006 and living in Rotterdam were invited to participate, resulting in 9778 mothers who participated in the study (24). The INMA project consists of 7 birth cohorts in Spain, 2 of which were included in the current study due to data availability: Sabadell (n = 657) and Gipuzkoa (n = 638), which enrolled pregnant women from July 2004 until July 2006 and April 2006 until January 2008, respectively. Ethical approval was obtained prior to recruitment from the Medical Ethics Committee of the Erasmus Medical Centre, Rotterdam (Generation R), Ethical Committee of the Municipal Institute of Medical Investigation, and the Ethical Committee of the hospitals involved in the study (INMA). Written informed consent was obtained from all participants and/or the children's parents or guardians.

For the current study, mother-child pairs were eligible if a Tg measurement was available. Further, either a maternal or cord blood TSH or free T4 (FT4) measurement, a child IQ measure, child brain magnetic resonance imaging (MRI) data or a UI/Creat measurement had to be available. Cord blood and MRI measurements were only available in Generation R.

Mother-child pairs were excluded in case of maternal Tg-antibody (Tg-Ab) positivity (defined as Tg-Ab > 40 IU/mL by manufacturer), a self-reported preexisting thyroid disorder, treatment for a thyroid disorder, a twin pregnancy, or in-vitro fertilization. In addition, participants with suboptimal child brain MRI data quality or a major incidental finding from analyses on related outcomes were excluded. We randomly excluded 1 of any sibling pair.

### Thyroglobulin and Thyroid Measurements

Tg, TSH, and FT4 concentrations were measured in serum samples obtained in early pregnancy (<18 weeks) and stored at −80 °C. Tg and Tg-Ab concentrations were measured at the ETH Zurich (Zurich, Switzerland) using an immunoassay calibrated using manufacturer standards (IMMULITE, Siemens Healthcare Diagnostics, UK, RRID for Tg: AB_2756379). The coefficients of variation for Tg concentrations were 9.3% at 1.9 ng/mL (n = 35), 7.1% at 9.6 ng/mL (n = 35), and 7.6% at 58.8 ng/mL (n = 35). More details on Tg measurement can be found elsewhere (23).

TSH and FT4 were measured using chemiluminescence assays (Vitros ECI; Ortho Clinical Diagnostics; Rochester, NY, USA) in Generation R and a solid-phase, time-resolved sandwich fluoro-immunoassay (AutoDEL-FIA, PerkinElmer Life and Analytical Sciences, Wallac Oy, Turku, Finland) and a lanthanide metal europium label in INMA. Cord blood TSH and FT4 were measured in serum samples obtained directly after birth using the same assay as maternal TSH and FT4.

Hypothyroxinemia (normal TSH and low FT4), subclinical hypothyroidism (high TSH and normal FT4), and subclinical hyperthyroidism (low TSH and normal FT4) were defined according to the 2.5th and the 97.5th population-based percentiles of thyroid peroxidase antibodies (TPOAb) negative (<60 IU/mL) and TgAb-negative women in Generation R and of TgAb-negative women in INMA, because TPOAb measurements were not available in this cohort. The reference group consisted of euthyroid women (TSH and FT4 between the 2.5th and 97.5th percentiles). The percentiles were defined in women with available TSH and FT4 measurements without selection on availability of neurodevelopmental outcomes.

### Urinary Iodine Measurements

Spot urine samples were collected at study visits during early pregnancy (<18 weeks) and stored at −20 degrees. In these samples, creatinine concentrations were measured using the Jaffe rate method (25), and iodine concentrations were measured using the Sandell–Kolthoff method in Generation R and using paired-ion reversed phase, high-performance liquid chromatography with electrochemical detection at a silver working electrode (Waters Chromatography, Milford, MA, USA) in INMA. Iodine calibration was performed using certified reference materials Seronorm urine levels 1 and 2 (Nycomed, Norway) and 4 EQUIP samples certified for urinary iodine concentration (Centers for Disease Control and Prevention, Atlanta, GA, USA). To account for urine dilution, iodine concentration was divided by creatinine concentrations to give the UI/Creat (in µg/g). A more detailed description of the urine collection and analysis of iodine concentrations can be found elsewhere (9, 26).

### IQ Measurements

If available, total IQ was used as the most complete measure with the most variance. Nonverbal and verbal IQ were separately assessed in secondary analyses when available. In Generation R, nonverbal IQ was assessed at a median age of 6.2 years using a subset of the Snijders Oomen Nonverbal Intelligence Test (2.5-7-Revised) (27) and total IQ at a median age of 13.6 years using 4 subscales of the Wechsler Intelligence Scale for Children, fifth edition (WISC-V), including Matrix Reasoning, Coding, Digit Span, and Vocabulary (28, 29). The Matrix Reasoning and Vocabulary subscales were used as measures of nonverbal IQ and verbal IQ, respectively. In INMA, total, nonverbal, and verbal IQ were assessed at a median age of 4.5 years using McCarthy Scales of Children´s Abilities (30) and nonverbal IQ at a median age of 8.5 years using 2 subscales of the WISC-IV including Coding and the Symbol Search subtest (31). All measurements were performed by trained staff. To homogenize the different scores, raw cohort-specific scores were standardized to a mean of 100 and an SD of 15. Children with IQ scores <50 or >150 were censored to 50 (n = 7) or 150 (n = 1), respectively. IQ measurements were combined into early childhood IQ (age 4.5 years in INMA and 6 years in Generation R) and late childhood IQ (age 9 years in INMA and 13 years in Generation R).

### MRI Outcomes

Before scanning, children at 10 years of age were familiarized with the scanning environment in a mock scanning session. MRI scans were obtained with a Discovery MR750w 3.0T scanner (GE Healthcare, Milwaukee, WI, USA) using an 8-channel head coil. T1-weighted images were acquired with an inversion recovery prepared fast spoiled gradient recalled sequence. Cortical reconstruction and volumetric segmentation were performed with FreeSurfer, version 6.0.0 (32). The quality of FreeSurfer output was visually inspected, and all scans with insufficient quality were excluded from statistical analyses (33). Brain volumetrics were extracted (eg, total gray matter volume and cerebral white matter volume), details of which have been described elsewhere including extensive quality control (33, 34). These global measures were chosen as primary brain morphology outcomes since region-specific effects were not currently hypothesized but were considered as possible follow-up analyses.

### Covariates

Potential confounders were selected a priori based on previous studies of the association of iodine concentrations with neurodevelopment outcomes (6) and biological plausibility. Information on maternal age at enrollment, national origin (cohort-specific categories), parity (0, 1, or >1), smoking behavior (no smoking during pregnancy, smoked until pregnancy recognized, and continued smoking during pregnancy), and highest achieved education (high, intermediate, low) was obtained through questionnaires filled in during pregnancy. Midwives and hospital registries provided information on child sex. Gestational age at blood sampling was defined using ultrasonography. Gestational age at birth, child age at MRI scan, or child age at IQ measurement were included as independent predictors of cord blood thyroid hormone concentrations or neurodevelopmental outcomes.

### Statistical Analyses

Descriptive characteristics of mother-child pairs in the analyses were compared to those without outcome data. Tg values above twice the upper reference value (ie, >110 ng/mL) as defined by the laboratory in which Tg was measured were excluded beforehand (n = 21) because of potential outlier effects. Outliers of other measurements were excluded beforehand based on visual inspection of the distribution of the untransformed variable: maternal TSH >15 mU/L (n = 3), maternal FT4 >45 pmol/L (n = 3), cord blood TSH >70 mU/L (n= 4), and UI/Creat >1000 µg/g (n = 11). Tg values were log-transformed when studied as a dependent variable to normalize residuals or when TSH or FT4 where the dependent variable to make the association linear. TSH values were log-transformed when studied as a dependent variable, and gestational TSH and FT4 were transformed to SD scores to make concentrations comparable across cohorts.

First, multivariable linear regression models were used to study the associations of Tg with maternal or newborn TSH and FT4 concentrations. Next, associations of Tg with offspring IQ and brain morphology were assessed. Two models were constructed: model 1 (adjusted for cohort) and model 2 (adjusted for cohort-specific national origin, gestational age at blood sampling, maternal age, BMI, smoking, parity, education, and child sex). Cord blood, IQ, and MRI analyses were also adjusted for (gestational) age at outcome measurement. To assess nonlinearity, a squared term for Tg was added to the models. If this indicated nonlinearity and improved the model fit, associations were visually inspected using a model with a restricted cubic spline with 3 knots. Results from the 2 cohorts were meta-analyzed using a 1-step approach. Since using a random intercept for cohort or adjusting for cohort-specific national origin provided similar results, the latter approach was chosen for simplicity.

Several additional analyses were performed. First, we studied the association of UI/Creat with Tg. Second, effect modification by UI/Creat was assessed by adding a product-interaction term of Tg with UI/Creat to model 2 with thyroid function or neurodevelopment as outcomes. In case of any indication of effect modification, subsequent stratification by UI/Creat according to a cut-off often used to distinguish iodine deficiency (<150 μg/g) from iodine sufficiency (>150 μg/g) (6) was performed. These analyses were planned based on the hypothesis that the combination of a low or high Tg with a low or high UI/Creat could be a better marker of iodine availability than each marker on its own and therefore associations of Tg with the studied outcomes might be stronger for low UI/Creat as compared to high UI/Creat. Third, any discovered associations of Tg with neurodevelopment were adjusted for UI/Creat, TSH, or FT4 to assess the independence of Tg and these measures. Fourth, we aimed to perform a formal mediation analysis by thyroid function conditional on the presence of associations between (1) Tg and thyroid function, (2) Tg and neurodevelopment, and (3) thyroid function and neurodevelopment in the sample with available Tg measurements. Fifth, associations were adjusted for inverse probability of attrition weights. Inverse probability weighting based on maternal age, ethnicity, BMI, smoking, parity, education, alcohol use, marital status, household income, and child sex was used to correct for loss to follow-up and to make the findings more generalizable to the whole cohort. Weights above the 99th percentile were truncated. Sixth, participants with TgAbs above the limit of detection ( = 20 IU/mL) were additionally excluded to rule out any interference in the Tg measurements by TgAbs below the manufacturer cut-off of 40 IU/mL. Lastly, we performed a post hoc analysis in which we assessed potential effect modification by TPOAb status for neurodevelopmental outcomes in Generation R by adding a product-interaction term of Tg with continuous TPOAbs or TPOAb status (above vs below 60 IU/mL).

Missing covariate data was imputed 25 times with the Multivariate Imputation by Chained Equations method (35). UI/Creat, Tg, TSH, and FT4 concentrations and IQ and MRI measurements were included as predictors for the imputation but were not imputed. Except for maternal national origin (4% in Generation R and 0.4% in INMA), smoking (11% in Generation R and 7% in INMA), and education (8% in Generation R and 0.5% in INMA), missing data on covariates were less than 1%. All statistical analyses were performed with R statistical software version 4.2.1.

## Results

**Figure 1.**
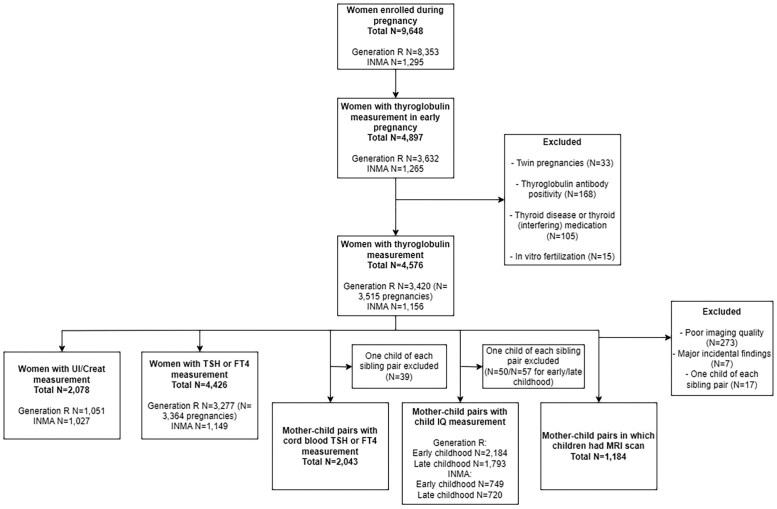
Flowchart of the study population.

The total study population comprised n = 4576 mother-child pairs with a range for availability of outcomes of n = 1184–4426 (Fig. 1). Descriptive statistics of the study population are shown in Table 1. Mean (SD) gestational age at blood sampling was 13.5 (2.0) weeks in Generation R and 13.5 (1.4) weeks in INMA. Median Tg concentrations were 11.1 ng/mL in Generation R and 11.6 ng/mL in INMA, whereas median UI/Creat was 208.9 μg/g in Generation R and 142.4 μg/g in INMA.

**Table 1.** Descriptive characteristics of the study population

	Generation R (n = 3515)	INMA (n = 1156)
Maternal characteristics		
Age, years, mean (SD)	29.7 (5.0)	31.5 (4.1)
BMI, Kg/m^2^, median (IQR)	23.5 [21.4, 26.5]	22.4 [20.8, 24.8]
Parity, n (%)		
0	2028 (57.7)	633 (54.8)
1	1041 (29.6)	440 (38.1)
≥2	446 (12.7)	83 (7.2)
Smoking, n (%)		
No smoking during pregnancy	2496 (71.0)	826 (71.5)
In the beginning of pregnancy	332 (9.4)	161 (13.9)
Continued during pregnancy	687 (19.5)	169 (14.6)
National origin, n (%)		
Spanish	—	1056 (91.3)
Latin American	—	69 (6.0)
Other	—	31 (2.7)
Dutch	1838 (52.3)	—
Moroccan	199 (5.7)	—
Turkish	298 (8.5)	—
Surinamese	316 (9.0)	—
European	288 (8.2)	—
Other Western	114 (3.2)	—
Other non-Western	462 (13.1)	—
Education, n (%)		
High	1536 (43.7)	450 (38.9)
Intermediate	1068 (30.4)	460 (39.8)
Low	911 (25.9)	246 (21.3)
Gestational age at blood sampling, weeks, mean (SD)	13.5 (2.0)	13.5 (1.4)
Thyroglobulin, ng/mL, median (IQR)	11.1 [7.0, 17.1]	11.6 [6.9, 18.8]
TSH, mU/L, median (IQR)	1.3 [0.8, 2.0]	1.2 [0.8, 1.8]
FT4, pmol/L, median (IQR)	14.8[13.2, 16.7]	10.4 [9.5, 11.3]
Urinary iodine to creatinine ratio, μg/g, median (IQR)	208.9 [139.3, 306.2]	142.4 [89.7, 236.8]
UIC, μg/L, median (IQR)	166.1 [97.3, 285.8]	128.5 [74.0, 214.0]
Child characteristics		
Male sex, n (%)	1790 (50.9)	591 (51.1)
Gestational age at birth, weeks, mean (SD)	39.9 (1.9)	39.7 (1.5)
Cord blood TSH, mU/L, median (IQR)	9.5 [6.5, 14.5]	—
Cord blood FT4, pmol/L, median (IQR)	20.7 [18.7, 22.8]	—
Age at IQ test early childhood, years, mean (SD)	6.2 (0.5)	4.5 (0.1)
IQ early childhood, mean (SD)	101.1 (15.3)	101.2 (14.5)
Age at IQ test late childhood, years, mean (SD)	13.6 (0.4)	8.5 (0.8)
IQ late childhood, mean (SD)	102.1 (13.9)	101.0 (14.7)
Age at MRI scan, years, mean (SD)	10.2 (0.7)	—

Data are shown after multiple imputation (see Methods section).

Abbreviations: BMI, body mass index; FT4, free T4; IQR, interquartile range; UIC, urinary iodine concentration.

In nonresponse analyses, Tg concentrations did not meaningfully differ between mother-child pairs with and without outcome data availability, although women included in the analyses were more often Dutch (Generation R) or Spanish (INMA), had a higher mean age, were less likely to smoke during pregnancy, and were more often highly educated compared to those of mother-child pairs without outcome data (Supplementary Table S1 and Supplementary Table S2) (36).

### Maternal and Newborn Thyroid Function

**Figure 2.**
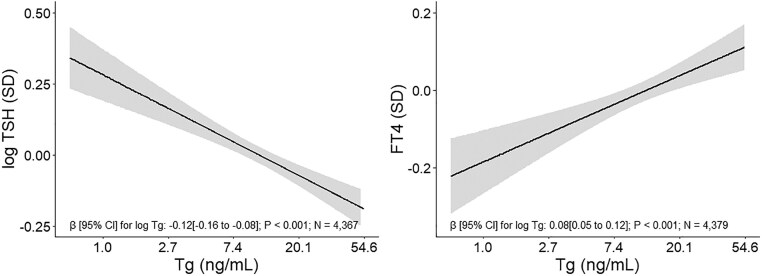
Associations of thyroglobulin with maternal thyroid function. Associations are adjusted for national origin (cohort-specific categories), gestational age, maternal age, education, BMI, parity and smoking, and child sex. Tg (ng/mL) and TSH (mU/L) are transformed by the natural logarithm. Back-transformed values for Tg are shown on the x-axis for better interpretation. TSH (mU/L) and FT4 (pmol/L) are standardized. Abbreviations: BMI, body mass index; FT4, free T4; Tg, thyroglobulin.

There was a negative association of Tg with TSH (β [95% confidence interval (CI)]: −0.12 [−0.16 to −0.08]) and a positive association of Tg with FT4 (β [95% CI]: 0.08 [0.05 to 0.12]) during pregnancy (Fig. 2 and Supplementary Table S3) (36). Findings for (sub)clinical thyroid disease entities were in line with the continuous associations. Higher Tg was associated with a lower risk of hypothyroxinemia (odds ratio [OR] [95% CI]: 0.78 [0.61 to 1.00]) and subclinical hypothyroidism (OR [95% CI]: 0.68 [0.56 to 0.83]) but with a higher risk of subclinical hyperthyroidism (OR [95% CI]: 1.55 [1.11 to 2.17]) (Supplementary Table S4) (36). There was no association of Tg with cord blood TSH (β [95% CI]: −0.02 [−0.06 to 0.01]) or FT4 (β [95% CI]: 0.01 [−0.19 to 0.21] (Supplementary Table S5) (36).

### Offspring IQ and Brain Morphology

**Figure 3.**
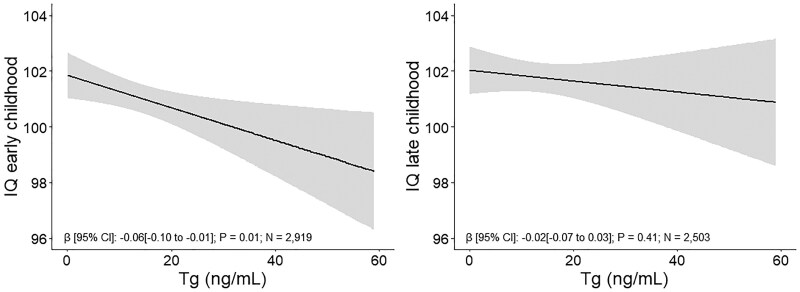
Associations of thyroglobulin with IQ. Associations are adjusted for national origin (cohort-specific categories), gestational age, maternal age, education, BMI, parity and smoking, and child sex and age at IQ measurement. Early childhood IQ includes IQ measured at 6.2 years in Generation R and 4.5 years in INMA. Later childhood IQ includes IQ measured at 13.6 years in Generation R and 8.5 years in INMA. Abbreviations: BMI, body mass index; INMA, Infancia y Medio Ambiente.

Higher Tg was associated with a lower IQ in early childhood (total IQ in INMA at age 4.5 years and nonverbal IQ in Generation R at age 6 years) (β [95% CI]: −0.06 [−0.10 to −0.01]) (Fig. 3 and Supplementary Table S6) (36). When cohorts were analyzed separately, this association was only present in Generation R. We found no association of Tg with late childhood IQ (nonverbal IQ in INMA at age 9 years and total IQ in Generation R at age 13 years) (Fig. 3 and Supplementary Table S6) (36). In secondary analyses, nonverbal and verbal IQ were studied instead of total IQ if available (Supplementary Table S7) (36). Tg was not associated with nonverbal IQ in early childhood (β [95% CI]: −0.04 [−0.09 to 0.00]) or late childhood (β [95% CI]: −0.02 [−0.07 to 0.04]). Tg was also not associated with verbal IQ in early childhood (β [95% CI]: −0.02 [−0.10 to 0.05], only available in INMA) or in late childhood (β [95% CI]: 0.00 [−0.06 to 0.06], only available in Generation R).

There was no relationship between maternal Tg and total gray matter volume (β [95% CI]: 0.14 [−0.15 to 0.44]) or cerebral white matter volume (β [95% CI]: 0.07 [−0.16 to 0.31]) at age 10 years (Supplementary Table S8) (36).

### Additional Analyses

Lower UI/Creat was associated with a higher Tg (β [95% CI] for 100-fold log Tg: −0.03 [−0.06 to −0.01]), and no nonlinearity was observed (Supplementary Fig. S1 and Supplementary Table S9) (36). There were no signs of effect modification by UI/Creat for any of the associations with thyroid function, IQ, or brain morphology outcomes (Supplementary Table S10) (36). Although FT4 was nonlinearly associated with early childhood IQ in Generation R, this association disappeared when restricting the sample to mother-child pairs with an available Tg measurement (Supplementary Table S11 and S12) (36). Hence no mediation analysis was conducted. Adjustment for UI/Creat, maternal TSH, or FT4 left the association of Tg with early childhood IQ essentially unchanged (Supplementary Table S13) (36). This also suggests that there would have been no meaningful mediation effect by thyroid hormones. Correction for inverse probability of attrition weights did not change the results (data not shown). Likewise, results remained similar when participants with TgAbs >20 IU/mL were excluded (Supplementary Table S14) (36). Our post hoc analysis did not show any effect modification by TPOAb status for associations of Tg with neurodevelopment (Supplementary Table S15) (36).

## Discussion

In this meta-analysis of 2 population-based prospective cohorts, Tg was associated with a lower maternal TSH and a higher maternal FT4. Tg was also associated with a lower IQ in early childhood but not with IQ or brain morphology in late childhood. Moreover, UI/Creat did not modify any associations of Tg with thyroid function or neurodevelopmental outcomes.

Both low and high maternal FT4 concentrations during pregnancy were associated with lower offspring IQ in a previous study in Generation R (37), although in INMA and another cohort this was found for low FT4 only (38). In addition, there was a positive association between UI/Creat and verbal IQ in partially the same study sample (9). Therefore, our finding that a higher Tg was associated with a lower early childhood IQ could partially be a reflection of either a lower iodine status or a higher FT4 concentration. Unfortunately, the lack of an association between thyroid function and neurodevelopment in the sample with available Tg measurements did not allow for a formal mediation analysis. Our study thus provides no support for the notion that the association of Tg with early childhood IQ may be mediated through thyroid function. In addition, these results remained essentially unchanged after adjustment for FT4, TSH, or UI/Creat indicating that the variance in IQ explained by Tg does not overlap with that of UI/Creat. Large studies with sufficient overlap in these measures are required to further elucidate the complex interplay of these biomarkers in relation to neurodevelopment.

Although our finding that a higher Tg was associated with a higher FT4 is in line with previous studies (21, 22), this is counterintuitive when considering Tg as a marker for iodine status and aligns more with the concept of Tg as a marker for thyroid hormone synthesis. Our result of an association between lower UI/Creat and higher Tg replicates that of a meta-analysis that used UIC instead of UI/Creat (20) and suggests that a higher Tg mostly reflects a lower iodine status. Furthermore, the effect estimate of the association of UI/Creat with Tg was small and predominantly driven by the INMA cohort. The lack of association in Generation R could be partially explained by the overall sufficient iodine status, although findings were similar when restricting to women with UI/Creat <150 μg/g (data not shown). The nonevident nature of this association is also reflected by the fact that a previous study using the same 2 cohorts did not find an association of continuous UI/Creat with Tg, which was likely due to small differences in study sample selection and the exclusion of outliers in the present study (23). Importantly, a strong relationship between UI/Creat and Tg might not be expected since UI/Creat is not suitable for individual assessment and the iodine intake timeframe that it reflects is different than for Tg. Interestingly, differences in Tg concentrations were found when women were grouped into iodine deficiency and iodine sufficiency (23). Overall, we cautiously conclude that Tg might better reflect thyroid function than iodine status.

The association with IQ in early childhood but not with IQ in later childhood could be explained in various ways. First, the associations of interest might attenuate due to the progression of time and/or neuroplasticity. Other (postnatal) factors that affect brain development and function might become increasingly important or the initial suboptimal developmental outcomes might be overcome. Second, IQ in early childhood was ascertained using different IQ tests than in later childhood, and therefore it is possible that any true biological differences are differently quantified due to the test characteristics rather than the age of testing. Early childhood IQ was measured with a subset of the Snijders Oomen Nonverbal Intelligence Test in Generation R and McCarthy Scales of Children´s Abilities in INMA, whereas later childhood IQ was measured with subscales of the WISC-V in Generation R and WISC-IV in INMA. These differences in IQ tests hamper a direct comparison between the different ages.

When using nonverbal IQ in both cohorts at both time points in secondary analyses to increase comparability and assess the robustness of the findings, Tg was no longer associated with IQ in early childhood. In addition, no association between Tg and brain morphology was found in the current study. Although the latter could be partially explained by the smaller sample size compared to the IQ analyses, these findings do not imply consistent associations of Tg with neurodevelopment.

To our knowledge, this is the first study that has investigated the association of Tg concentrations during pregnancy with offspring neurodevelopment. We were able to perform this study in a large population-based sample of 2 cohorts with different iodine status and with detailed data on possible confounders. A limitation of the study is the use of a single Tg and UIC measurement, which did not allow for studying changes in Tg in relation to IQ and brain morphology. UIC has a high intraindividual and day-to-day variability, which can be reduced by creatinine adjustment as the correlation with measured 24-hour urinary iodine excretion is stronger for UI/Creat than for UIC (39-41). However, multiple spot samples of UI/Creat preceding the Tg measurement might provide a better indication of iodine status. Another limitation is that IQ was assessed with different tools in the 2 cohorts, although scores were standardized to facilitate comparisons. Lastly, the 2 studied cohorts were on average either iodine-sufficient or only mildly iodine-deficient. Therefore, the current study results are not easily generalizable to iodine-deficient populations. Associations between Tg and neurodevelopment might be more prominent in the case of iodine deficiency, although Tg might then still reflect thyroid function more than iodine status.

In conclusion, this study shows that a higher Tg is associated with higher thyroid function during pregnancy and lower offspring IQ in early childhood but not with IQ or brain morphology in late childhood. In addition, our results do not provide evidence for an additional value of combined assessment of Tg and UI/Creat for associations with thyroid function or neurodevelopment. Although we show that Tg is related to 1 important neurodevelopmental outcome, Tg might reflect thyroid function better than iodine status, and its added value over measuring thyroid function itself is questionable. Further studies should replicate our findings in order to determine the added value of Tg in addition to UIC for defining iodine status.

## Data Availability

Some or all datasets generated during and/or analyzed during the current study are not publicly available but are available from the corresponding author on reasonable request.
